# Statistical Researches Relative to the Etiology of Pulmonary and Rheumatic Diseases

**Published:** 1842-04

**Authors:** 


### Art. X.

*Statistical Researches relative to the Etiology of Pulmonary and Rheumatic Diseases, illustrating the application of the Laws of Climate to the Science of Medicine; based on the Records of the Medical Department and Adjutant-General's Office.* By Samuel Forry, m.d., Medical Staff, United States Army, &c. (Reprinted from the American Journal of the Medical Sciences.)—*Philadelphia*, 1840. 8vo, pp. 44.

The attention of the writer of this essay was first directed to the subject of it whilst arranging the Army Meteorological Register, and the Medical Statistics of the American Army, which we are glad to find are now in course of publication. In addition to the object announced in the title-page, the author wishes "to demonstrate the advantages of peninsular Florida as a winter residence for pulmonic invalids."

Few climates promise greater facilities for solving such questions as are here proposed, than that of the United States. "The extreme north," we are told, "has a climate in which cold predominates, vexed by winds that have passed over interminable snows; the south acknowledges the genial influence of the sun; whilst the middle vibrates alternately to both extremes. The climate of the United States is, in truth, remarkably inconstant and variable, "passing rapidly" says Malte-Brun, "from the frosts of Norway to the scorching heats of Africa, and from the humidity of Holland to the drought of Castille." So sudden are the vicissitudes of weather in the middle States, that, in the language of the Spectator, we often "lie down in July and rise in December." In a note we are told that "the climate of the new world, viewed in its general feature, is, contrary to common opinion, more mild and uniform than that of the old. Taking, for example, the annual temperature of 53°·60, the eastern coast of Asia, shows a difference of 55°·80 in the mean temperature of summer and winter, whilst the eastern coast of America exhibits a difference only of 43°·60; and, on the other hand, the western coasts of Europe and of America present respectively a difference of 28°·30 and 23°. It appears too that the western coast of North America has a lower mean temperature than the western coast of Europe under the same latitude. Thus Bordeaux, which is on the same parallel with Fort Sullivan, has an annual temperature of 15° higher. The lower temperature of the North American continent is attributable doubtless to the circumstance of the warmer parts of America being continuous with the frozen regions of the north, whilst Europe is separated from those regions by intervening seas.

The facts, therefore, contained in this essay refer to an extensive country presenting two strongly contrasted regions, a north and a south, and an intermediate one made up of the other two, and presenting alternately the characters of both. The *climate* of this and of other countries is made up of many elements which it would be very difficult to submit to separate measurement. Accordingly only two are selected for examination, *the temperature of the air and its hygrometric state*—"agents always supposed to exert a potent influence in the causation of pulmonary and rheumatic diseases." For the purpose of this enquiry the posts occupied by troops are divided into three groups—a northern, middle, and southern; the northern comprising posts on the coast of

New England, extending as far south as the harbour of New York; posts on the northern chain of lakes, and posts remote from the ocean and inland seas; the middle, the atlantic coast, from Delaware Bay to Savannah, and interior stations; and the southern, the posts on the Lower Mississippi, and in the peninsula of East Florida. These grand divisions not only present the differences already mentioned among themselves, but the subdivisions present peculiarities of climate which must not be neglected. Thus we are told that "the northern division presents the greatest variety of climate; on the sea-coast of New England the influence of the ocean modifies the range of the thermometer and the mean temperature of the seasons. Advancing into the interior the extreme range of temperature increases, and the seasons are violently contrasted. Having come within the influence of the great lakes, a climate like that of the sea-board is found, and proceeding into the region beyond the modifying agency of those inland seas, an excessive climate is again exhibited." A table is subjoined in which the temperature of the sea-coast is contrasted with that of the posts remote from the ocean and inland seas. From this table it appears that the mean temperature of the winter on the sea-coast is 6°·05 higher than in parts remote from the ocean and inland seas; the temperature of the spring, however, is lower by 4°·13, that of the summer by 8°·71, and that of the autumn by 0°·40. A more striking contrast is afforded by comparing the difference between the mean temperature of summer and winter in the two districts respectively. On the sea-coast the difference is 38°·61, and remote from the coast 53°·37. This fact shows that the mean annual temperature is not all that must be taken into account in comparing one locality with another, the extraordinary inequality presented in each by the seasons being at least as important an element of climate. A similar comparison to the above is also presented for posts on the lakes, and posts remote from the lakes, but in the same region, from which it appears that whilst the mean temperature of the winter near the lakes, (which lakes are 1°·46 north of the posts remote from them,) is 2°·54 higher; that of the summer is 10°·40, that of the spring 6°·65, and that of the autumn 2°·04 lower. The difference between the mean temperature of summer and winter on the lakes is 43°; that of the districts remote from them 55°·84. Another striking difference between the two localities is in the number of fair, cloudy, rainy, and snowy days. On the lakes the number of fine days is little more than half those in the remoter districts, the number of cloudy days nearly double, the number of rainy days is as 7 to 5 nearly, and that of days on which snow falls, 5 to 3 nearly. From this comparison it appears that on the shores of the ocean, or inland seas, the temperature is more equable, the air more moist, the changes of season more slow, uncertain, and variable; and that on the other hand, the climate of places remote from the seas and lakes is characterized by a great range of temperature, a strong contrast between winter and summer, and constant and rapid changes of season. The districts of the sea-shores and of the lakes resemble each other sufficiently to admit of being classed together, that of the district remote from both presents remarkable characters. "From November to May, cold weather prevails, the ground being generally covered with snow to the depth of three or four feet, and the general range of the thermometer being from the freezing point to 30° below zero. The summers are equally remarkable for extremes of temperature. The heat is often as oppressive as in Florida, the mercury sometimes rising in June, July, and August, to 100° Fahrenheit in the shade. Unlike the two preceding classes, in which the air is moist, and the changes of the seasons more slow and uncertain in this one, a constant and rapid succession is observed among the seasons, summer succeeds winter so rapidly that there is scarcely any spring, and vernal vegetation is developed with remarkable suddenness."

In the *Middle Division* we are told that the two systems of climate bear the same meteorological relation towards each other, as the third class of the *Northern Division* does to the first two. "The *Southern Division* is characterized by the predominence of high temperature. Proceeding south from Canada to Florida, the seasons become more uniform in proportion as their annual temperature increases. This is strikingly illustrated on comparing the difference between the mean temperature of summer and winter at Fort Snelling, Iowa, and at Key West, at the southern point of Florida, the former being 55°·51, and the latter only 11°·34." Our space will not allow us to enter into more minute accounts of the climate of the several localities contrasted;—the most important features have been already stated. We now come to the tables which present the influence of those strongly contrasted localities on the health of the troops. The aggregate mean strength employed amounts to 47,220, and the period of observation extends from 1829 to 1838 inclusive. In the formation of the tables 1500 quarterly reports of sick have been used, and the mean strength of each post has been computed from monthly returns in the adjutant-general's office.

The army of the United States is comparatively small, the whole force not exceeding 5000 men; and as these are scattered over an immense extent of territory, and subject to every variety of climate and temperature, the mortality may be expected to differ very considerably in different parts of the Union. Accordingly we find that, while in the northern division it does not exceed 15 per 1000, or about the same proportion as among British troops in North America, the average in the southern and middle division of the States is as high as 42 per 1000 annually. Indeed, at some of the southern stations, particularly those on the Lower Mississippi, the loss of the troops has been as great as in several of the West India Islands during the same period, 53 per 1000 having perished annually. Even in East Florida, of which as a residence for invalids Dr. Forry draws so flattering a picture, the mortality on the average of ten years was 39 per 1000; and in 1836 and 1837 it amounted to double that rate.

Of the diseases by which the mortality in the United States Army has been occasioned, no specific information is given, except as regards pulmonic and rheumatic affections; and it must be premised that the deductions, even as to these, are liable to considerable exception, owing to the causes of death having in many instances been omitted. Defects of this kind, however, are not likely to be so common in reporting the cases which came under treatment, because the stoppage to which the soldier is subject when in hospital, and which we presume exists in the American as well as the British army, must operate as a check upon any omission.

The following Table exhibits a summary of the Returns on which Dr. Forry has founded his conclusions in regard to the influence of climate in producing pulmonic affections in different parts of the States:

| | Aggregate mean strength on average of 10 years. | Total admissions into Hospital in 10 years. | | | | Ratio of admissions per thousand of mean strength annually. | | | | Total deaths in 10 years from | | | |
|---|---|---|---|---|---|---|---|---|---|---|---|---|---|
| **Northern Region.** | | Catarrh and Influenza. | Pneumonia. | Pleuritis. | Phthisis Pulmonalis. | Catarrh and Influenza. | Pneumonia. | Pleuritis. | Phthisis Pulmonalis. | Catarrh and Influenza. | Pneumonia. | Pleuritis. | Phthisis Pulmonalis, and Hemophysis. |
| Atlantic Posts......... | 3663 | 760 | * 68 | * 82 | 38 | 208 | 22 | 26 | $10\frac{4}{10}$ | ... | 1 | ... | 15 |
| Posts on the Lakes ... | 5976 | 1801 | 111 | 181 | 50 | 300 | 19 | 30 | $8\frac{4}{10}$ | 1 | 4 | ... | 9 |
| Posts remote from the Ocean and Lakes ............ | 12604 | 6977 | 211 | 355 | 65 | 553 | 17 | 28 | $5\frac{1}{10}$ | ... | 3 | 1 | 23 |
| Total...... | 22242 | 9538 | 390 | 618 | 153 | 429 | 18 | 28 | 7 | 1 | 8 | 1 | 47 |
| **Southern Region.** | | | | | | | | | | | | | |
| Coast from Delaware Bay to Savannah ............ | 5850 | 2842 | † 80 | † 102 | 65 | 486 | 25 | 32 | $11\frac{1}{10}$ | 4 | 1 | 1 | 36 |
| South Western Stations ...... | 11140 | 3233 | 430 | 592 | 123 | 290 | 39 | 52 | 11 | ... | 31 | 2 | 63 |
| Posts on the Lower Mississippi ...... | 3381 | 739 | 73 | 96 | 30 | 218 | 22 | 28 | 9 | ... | 2 | 2 | 10 |
| East Florida ......... | 4607 | 658 | 68 | 113 | 40 | 143 | 15 | 24 | $8\frac{7}{10}$ | ... | ... | 1 | 9 |
| Total...... | 24978 | 7472 | 651 | 903 | 258 | 299 | 26 | 36 | $10\frac{3}{10}$ | 4 | 34 | 6 | 118 |

We have given these results precisely as they are stated in an abstract appended to the pamphlet under review, merely omitting the cases of pneumonia and pleuritis at Fort Independence and Fort Monro on account of the probable sources of error pointed out by Dr. Forry; and we shall now proceed to consider how far they correspond with the conclusions drawn on the same subject from the Statistical Reports of the British Army by Major Tulloch, as the value of all these statistical documents depends on the facilities they offer for the purpose of comparison.

One of the most important and perhaps the most startling of the deductions drawn from the Statistical Reports of the British army, was the greater frequency of phthisis pulmonalis in the tropical regions of the west, than in our own cold, variable, and much-abused climate, or even in the still more inclement regions of Canada. Now, on referring to the influence of climate on this disease in the United States army, as shown in the preceding abstract, results precisely similar are obtained. Thus, throughout the whole of the southern divisions possessing a climate somewhat similar to Bermuda, and the most northerly of the West India

* These numbers are exclusive of the cases at Fort Independence.

† These numbers are exclusive of the cases at Fort Monro.

Islands, the proportion attacked annually by that disease averages $10\frac{3}{10}$ per 1000 of the mean strength, while throughout the northern region the average is only 7 per 1000 annually, and in that part of the northern region where the climate is the most inclement of all, the proportion attacked is but $5\frac{1}{10}$ per 1000. Compare this with the proportion attacked at each of the following stations, as shown by the Statistical Reports of the British army, viz.:

| | |
|---|---|
| In Jamaica . . . . . | 13 |
| West Indies . . . | 12 |
| Bermuda . . . . | 9 |
| Canada . . . . . | $6\frac{1}{2}$ |
| United Kingdom . . | $6\frac{1}{2}$ |

The returns of both armies will be found to establish most clearly and distinctly the same principles, viz., that phthisis is nearly twice as prevalent in the southern as in the northern regions of the western hemisphere, though the nature of the climate and our preconceived notions of the agencies by which that disease is influenced would *à priori* have led us to anticipate the reverse conclusion.

With his own tables before him to prove the contrary, we are surprised to find Dr. Forry stating (p. 22,) that phthisis, pneunomia, and pleuritis are less common in the peninsula of Florida than in any other part of the United States. That this is not the case as regards the first of these diseases at least, will at once be manifest on reference to the abstract on the preceding page, where the proportion attacked is shown to have been

| | | |
|---|---|---|
| | $8\frac{7}{10}$ | per 1000 annually. |
| While on the posts on the northern lakes *it was only* . . . . . . . . . . | $8\frac{4}{10}$ | " " |
| Posts remote from the ocean and lakes, *only* | $5\frac{1}{10}$ | " " |

So that the climate of Florida, to which Dr. Forry is so anxious to send consumptive patients, absolutely by his own figures is proved to originate more than an average number of cases; to say nothing of an annual mortality of 61 per 1000 from other diseases, and the chance of death from causes not within the province of the doctor, such as being scalped by the Seminole Indians, an event we believe by no means uncommon in that quarter.

Dr. Forry appears equally unfortunate in his deductions in favour of Florida as a residence for consumptive patients, if the mortality by that disease as deduced from the preceding abstract is taken as the criterion: for throughout the northern regions

| | | |
|---|---|---|
| This amounted annually to . . | 2·1 | per 1000 of the strength. |
| In Florida, to . . . . . . . . | 2·0 | " " |
| Throughout all the southern and middle stations, to . . . | 4·8 | " " |

So that the utmost which can be said of Florida is, that the mortality from phthisis is less than in the other southern or middle stations; but unquestionably it is much the same as in the north, which we presume, from Dr. Forry's train of reasoning, is the quarter whence he is most anxious to draw his consumptive patients to enjoy the balmy influence of a southern climate. Though we have arrived at these conclusions from the doctor's own figures we apprehend that, as in the British army, so large a proportion of the consumptive patients are discharged from the service before the disease has time to arrive at a fatal termination, that it is impossible to establish with certainty the exact proportion of deaths which it occasions at the different stations; but this source of error would of course be just as likely to affect the northern as the southern divisions.

The following Table made up from Dr. Forry's returns affords, however, still better means of testing the advantage or disadvantage likely to be derived by a removal even in winter, from a northern to a southern climate:

| NORTHERN REGION. | Mean strength. 1st quarter. | Mean strength. 4th quarter. | Average of winter season. | Attacked by Phthisis. 1st quarter. | Attacked by Phthisis. 4th quarter. | Attacked by Phthisis. Total in winter season. | |
|---|---|---|---|---|---|---|---|
| Atlantic Posts................... | 4018 | 3377 | 3697 | 7 | 10 | 17 | or 4·6 per 1000. |
| Posts on the Lakes............ | 6370 | 6205 | 6287 | 17 | 10 | 27 | or 4·3 ,, |
| Posts remote from Ocean and Lakes ............... | 12837 | 13219 | 13028 | 28 | 14 | 42 | or 3·2 ,, |
| Total in Northern Regions | | | 23012 | 52 | 34 | 86 | or 3·7 ,, |
| Florida ......................... | 5134 | 4421 | 4777 | 11 | 9 | 20 | or 4·2 ,, |

From this comparison it is obvious that fewer cases originate throughout the northern States during the extreme severity of their winter than in the mild and equable winter of Florida, where the thermometer rarely falls below 50°. To remove a patient, even on the approach of winter, from the former to the latter would therefore, according to all the ordinary rules of arithmetic, increase the prospect of confirming or aggravating the disease in the proportion of 42 to 37, and even if, instead of the average, we had taken the worst of the northern stations as our standard, the chance of amelioration would have been only in the proportion of 46 to 42, too slight a difference certainly to warrant the high expectations entertained by Dr. Forry.

This conclusion does not rest merely on one series of facts: for the information supplied by the pamphlet under review shows that, out of the whole 412 cases in the 10 years under observation, the proportion which originated in summer and winter was as follows:

| | | The number attacked was | Out of an aggregate strength of |
|---|---|---|---|
| In the winter season. | 1st Quarter . . | 122 . . | 48976 |
| | 4th Quarter . . | 85 . . | 46608 |
| | | 207 | 47792 or $4\frac{4}{10}$ per thousand. |
| In the summer season. | 2d Quarter . . | 106 . . | 46310 |
| | 3d Quarter . . | 99 . . | 46981 |
| | | 205 | 46645 or $4\frac{3}{10}$ per thousand. |

Being, to within a fraction, precisely the same proportion in summer as in winter. Dr. Forry arrives at the same results by a different series of calculations, in which he shows the total ratios attacked by phthisis at all the stations to have been in each quarter as under:

| 1st Quarter. | 2d Quarter. | 3d Quarter. | 4th Quarter. |
|---|---|---|---|
| 19 | 19 | 15 | 15 |

The sum of the first and fourth quarters constituting the summer months is 34, and that of the second and fourth precisely the same; yet he states that no general laws "can be deduced from these numerical results," though there is this general and most important law standing out in broad relief before him that, as precisely the same proportion of attacks are found to originate in summer as in winter, and in the mildest regions of the south, as in the coldest and most inhospitable of the north, it is, to say the least, most improbable, that benefit can accrue in cases of genuine phthisis, by a removal from the latter to the former at any stage of the disease. Change of scene, change of air, and the excitement produced by travelling in a country which presents much novelty of character to interest the patient may always be expected to exert a cheering, and in some even a sanatory influence, but so far as can be determined by the arithmetical evidence before us, precisely the same result must ensue even if the transition was from a warm climate to a cold one. In this remark, however, we do not include cases of chronic bronchitis which, being more nearly allied to simple catarrhal affections may, like that class of diseases, be more susceptible of benefit from a mild and equable climate.

The circumstance stated by Dr. Forry, that "nearly all the cases of consumption in the northern division, are ascribed to the use of ardent spirits," would seem still further to diminish the benefit he anticipates from a southern residence, for how much greater must be the tendency to consumption there, when the cases are so much more numerous without that exciting cause to induce them. Though this view would materially favour our previous conclusions, we are not disposed to coincide with Dr. Forry in the belief that the relative prevalence of phthisis is materially affected by intemperance, knowing that the use of plantation rum is just as common in the southern, as "gin-sling" or "cocktail" in the northern divisions of the States; still less are we inclined to coincide with him in the supposition, that the prevalence of that disease in some southern latitudes is attributable to the increased susceptibility to chronic affections, and the debilitating heat in such climates. On the contrary we know from undoubted authority that in the British army in the East Indies, a body of men perhaps more addicted to the use of spirituous liquors than any in Her Majesty's service, and all suffering less or more from chronic disease, and the debilitating effect of the highest temperature to which the human frame is exposed, not two cases of genuine phthisis are found to occur among a thousand soldiers annually; and of those that do occur, a considerable proportion originated before the arrival of the individuals in that country.

One fact of this kind is worth volumes of argument, and affords in itself a sufficient reply to many of the objections stated by Dr. Forry, in ignorance of so remarkable a feature in the history of this disease. Before assuming the part of a critic, it is highly necessary in questions of this kind, to be fully informed of the influence of the disease not only in the western hemisphere, but also in the east; and we feel convinced if Dr. Forry will suspend his conclusions till he has all the facts fully before him, he will be satisfied on many points regarding which he at present appears sceptical.

Some will no doubt be disposed to put the question, why should such different results be obtained in the western compared with the eastern hemisphere, when the climate, so far as regards temperature at least, may be said very nearly to correspond? To such enquirers it may at present be a sufficient reply, that our business is, rather to state facts than to account for them; but surely there is nothing improbable in the conclusion that phthisis may, like many other diseases, have its favorite localities, and that the inhabitants of one quarter of the globe may be subject to its influence while those of another are comparatively exempt. Is there not before us the striking example of yellow fever, annually ravaging the southern shores of the United States and our West India islands, while in the same latitudes on the other side of the line, and under precisely similar circumstances of locality and temperature, not a case of the disease is found to occur,* and in the tropical regions of the east it is altogether unknown. Cannot the same power which has set limits to the pestilence, say also to consumption, thus far shalt thou come but no further? Or, because short-sighted mortals cannot as yet trace distinctly to physical agencies the wonders of his workings, are we to doubt the result of nearly a million of observations, extended for many years over thousands and ten thousands of our countrymen, and recorded by men whose professional character and zeal for science afford the best possible guarantee that they have been recorded with truth?

In regard to pneumonia and pleuritis, the facts exhibited in the returns of the American army quoted by Dr. Forry confirm, in the strongest manner, what had previously been ascertained in regard to the influence of these diseases in the Statistical Reports of the British Army. It was there shown that neither as regards prevalence nor severity did they seem to be at all influenced by temperature, moisture, or any of the known atmospherical agencies; that, in fact, the causes of their origin and operation were just as inexplicable as those of consumption. In Bombay, in Malta, in Nova Scotia and New Brunswick, climates so remarkably different, the proportion attacked on an average of 20 years was exactly 34 per thousand; and unfortunately for Dr. Forry's hypothesis, that "in proportion as the high temperature of summer makes an impression on the system, do the lungs become susceptible to the morbific agencies of the opposite seasons," it is found by the British Medical Returns, that the proportion attacked annually in Jamaica and Ceylon, where the difference between the temperature of summer and winter is only a few degrees, amounts to 14 and 18 per thousand respectively; while in the United Kingdom, where that difference is at least threefold as great, the proportion attacked is exactly a mean between the other two, or 16 per thousand. However pleasing it would be to discover the laws which regulate the operation of the causes of these diseases, we feel bound to call for stronger evidence than has yet been adduced by Dr. Forry before acceding to his conclusions.

* See Report on the Health of the Navy.

The following table given by Dr. Forry, to show the relative prevalence of these diseases at different seasons of the year, and in different parts of the Union, affords the strongest possible corroboration of the results and deductions on this head previously obtained from the Medical Returns of the British Army.

| | Ratio* treated per thousand of mean strength annually for pneumonia and pleuritis. | | | | | |
|---|---|---|---|---|---|---|
| | 1st quarter. | 2d quarter. | 3d quarter. | 4th quarter. | Annual results. | |
| NORTHERN DIVISIONS. | | | | | | |
| Posts on the Coast of New England ............ | 12 | 11 | 8 | 10 | 41 | average 45. |
| Posts on Northern Chain of Lakes ................ | 11 | 15 | 13 | 11 | 49 | |
| Posts north of lat. 39°, and remote from the ocean and inland seas ........................ | 14 | 11 | 7 | 12 | 45 | |
| Total Northern Division... | 37 | 37 | 28 | 33 | | |
| MIDDLE DIVISIONS. | | | | | | |
| Coast from Delaware Bay to Savannah ......... | 21 | 11 | 8 | 16 | 57 | |
| South-western Stations ............................ | 46 | 18 | 10 | 20 | 92 | |
| SOUTHERN DIVISIONS. | | | | | | |
| Posts on the Lower Mississippi ..... ........... | 20 | 9 | 4 | 11 | 47 | average 44. |
| Posts on the Peninsula of Florida.................. | 14 | 9 | 8 | 6 | 39 | |
| Total ............ | 138 | 84 | 58 | 86 | | |

Here it will be observed, that on the average of the three most northerly stations subject to great extremes of temperature, the proportion attacked is exactly 45; while on the average of the two southern ones, where the climate is comparatively mild and equable, the proportion attacked is exactly 44, as near an approximation as can possibly be looked for when fractions are rejected in the calculation. Again, with all that remarkable difference which exists in the Northern Divisions between the extreme heats of summer, when the thermometer often for months rises nearly to 100° of Fahrenheit, and the extreme cold of winter when it occasionally falls below zero, still we find from the preceding table that the proportion attacked there in the winter compared with the summer quarters was

| | | | |
|---|---|---|---|
| Winter Season | 1st Quarter . . | 37 | average 35. |
| | 4th Quarter . . | 33 | |
| Summer Season | 2d Quarter . . | 37 | average 32½. |
| | 3d Quarter . . | 28 | |

And not only does the average of the summer and winter attacks correspond thus nearly, but on the northern chain of lakes, the proportion attacked in summer is even considerably greater than in winter, the sum of the two winter quarters being only 22, while that

* The sum of the ratios in this table does not correspond exactly with the numbers given on page 426, owing to fractional parts being rejected, but we have stated it precisely as in Dr. Forry's pamphlet, p. 18.

of the two summer ones is 28. Yet along the southern shores of Lake Erie, Ontario, and Michigan, the summer is by no means short, and the temperature is high. We leave it to Dr. Forry to reconcile this simple fact with the law which he lays down, that in proportion as the high temperature of summer makes an impression on the system, do the lungs become susceptible of the morbific agency of the opposite seasons.

However much we may differ with Dr. Forry in his conclusions as regards other pulmonic affections we perfectly concur in what he states regarding catarrhal affections. The Statistical Reports of the British as well as those of the American army, all tend to the conclusion that these affections are generally most common where the seasons are violently contrasted, and that they are less dependent on diurnal variations of temperature than on their extreme range. In the returns from all the northern stations of the British army, the cases are nearly twice as numerous in winter as in summer, and on this account there can be little doubt that considerable benefit is likely to be derived by patients labouring under *chronic bronchitis*, provided they can exchange the extreme severity of a northern winter for the more genial influence of that season in the south. As regards consumption, pneumonia, and pleuritis, however, there exists no numerical evidence to warrant any such conclusions; on the contrary, all the facts, whether deduced from the returns of the American or the British army, clearly demonstrate the reverse.

It is impossible to say why such a distinction should exist in regard to the influence of the same causes on diseases hitherto supposed so nearly allied in their origin; all we can do is to notice the fact. It must always be kept in view that our knowledge of the geographical distribution of diseases is yet in its infancy, and that what little has been established is far from warranting much reliance on our preconceived notions as to the influence of climatorial agencies. Who, for instance, would have supposed that rheumatism, the absence or modified character of which was supposed to depend so materially on the mildness and equability of a climate, would be found more prevalent in the West Indies and Mauritius than in Nova Scotia or Canada? Yet it has been clearly established by the Statistical Reports of the British Army, that while the proportion attacked at the two latter stations was only from 30 to 40 per 1000 of the force annually, at the two former it was from 46 to 49 per 1000; and now we find by the Statistical Reports of the American Army, that in the southern division of the States, including the peninsula of Florida, the proportion attacked annually is as high as 119 per 1000 of the strength. That rheumatic affections are but little under the influence of the seasons, violently contrasted as these seasons are in most parts of the United States, is further shown by the following summary of the proportions attacked in each quarter, extracted from Dr. Forry's pamphlet.

| | | Attacked per thousand of strength. | |
|---|---|---|---|
| Winter Season | 1st Quarter | 249 | average 225. |
| | 4th Quarter | 232 | |
| Summer Season | 2d Quarter | 219 | average 210. |
| | 3d Quarter | 201 | |

The returns given by Dr. Forry show that rheumatic affections are exceedingly common throughout the American Army; the proportion of cases which come under treatment annually being from three to four times as high as in the British Army. He endeavours to account for this difference by the supposition that soldiers treated in quarters may have been omitted in the returns of the latter, which profess only to contain admissions into hospitals. In this respect, however, he is in error; as under that head is almost invariably included every case which comes under treatment, whether in hospital or in quarters, and the proportion of the latter is so small, that at the utmost it could not make more than a fractional difference in the results.

Having thus examined in detail the principal observations of Dr. Forry in regard to the etiology of pulmonary and rheumatic affections, we shall briefly notice some remarks by him, arising, we apprehend, principally from a want of acquaintance with the principles on which the British Statistical Reports are formed.

In the first place he objects to England having been assumed as the standard of comparison: now we conceive it would be difficult, even for the ingenuity of Dr. Forry, to devise any better standard for estimating the influence of various diseases among our troops in other countries, than by carefully observing, in the first place, to what extent they are liable to them in their own. A comparison of the extent to which the natives of each country are thus affected might, no doubt, have been exceedingly useful as a criterion in some diseases; but it would have been exceedingly deceptive as a standard in others. On the west coast of Africa, for instance, fevers cause only the same extent of mortality among the native troops as among troops in England, yet how enormous would be the conclusion were we from such data to assume that class of diseases to be comparatively rare there? It surely seems a much less exceptionable mode of investigation, to ascertain, in the first instance, the influence of each disease on a certain class of individuals in this country, and then to carry out the same series of observations on the same individuals, or others similarly circumstanced, throughout every quarter of the globe.

Besides, where are we to look for the means of comparing the diseases of the natives of different countries? Throughout all the stations embraced in the British Statistical Reports, no information of the kind has been attainable, except for Malta, and that, it must be admitted, the author has liberally availed himself of. Dr. Forry seems to think that a deduction should have been made of the deaths among children and old persons from marasmus, previous to drawing any conclusions as to the prevalence of consumption in that island, forgetting that even had it been practicable to make that separation, which it was not, it would have been necessary also, previous to drawing any conclusions, to separate from the strength the number of children and old persons who are liable to marasmus, and not to the more decided forms of consumption. This would have reduced the strength of the population about one half, and instead of diminishing the ratio of mortality by the latter disease would absolutely have increased it beyond what it was estimated at in the British Statistical Reports.

That diseases of the lungs create very nearly the same proportion of mortality among the civil population of Malta as among that of Great Britain, can now be very satisfactorily proved by the following calculation. The population of Great Britain at Midsummer, 1838, must have been, according to the late census, about 15,196,225, of whom, it is shown by the registrar-general's returns for that year, that 88,517 died of diseases of the lungs, (excluding hydrothorax, which is not inserted under that head in the Maltese returns,) being in the ratio of $5\frac{8}{10}$ per thousand annually, while the returns from Malta show that on the average of the last twenty years the mortality by the same description of diseases was $5\frac{1}{10}$ per thousand annually, a very close approximation it must be admitted, the more especially as 1838 was a year in which the mortality from diseases of the lungs was much increased in this country, owing to the very general prevalence of influenza. We have no doubt that a still nearer approximation will be obtained by comparing the deaths by this class of diseases in the registrar-general's returns for the year 1839, (now in course of publication,) with the returns from Malta for the same period, and in that view we shall take some future opportunity of submitting them to our readers.

Dr. Forry conceives it an error in the Statistical Reports of the British army, that the returns of the dragoon-guards and dragoons should have been assumed as the standard for estimating the relative influence of pulmonary disease in different countries. Had he been aware of the peculiar constitution of our army, he would have spared himself the trouble of so ill-founded an objection. To accurate statistical enquiry, it is essential that the individuals over whom it extends should be for a considerable period under observation. Now the regiments of the line being constantly in a migratory state, and seldom more than three or four years at home, would clearly have been a most objectionable standard, more particularly as a large proportion of the deaths occur from diseases contracted abroad. The depôts would have been still worse, because a constant interchange of healthy for unhealthy men is taking place between them and the service companies abroad. It was absolutely necessary, therefore, to take as the standard some class of troops permanently resident in this country. The only ones of this description are the foot-guards and cavalry, the former of whom being restricted to the duties of London, would clearly have been objectionable, as exhibiting, not the standard of health throughout the kingdom generally, but merely in the metropolis. It therefore became necessary to adopt the dragoon-guards and dragoons as the standard, being the only description of force which was not objectionable in these respects; and when it is kept in view that even among them the mortality by diseases of the lungs includes one half of all the deaths, it can certainly not be deemed too favorable a criterion of the influence of these diseases on troops in this country.

It would have been well for Dr. Forry to have made himself aware of all such particulars before he assumed the part of a critic on the admirable statistics of the British army: had this been the case, he would have saved much of the valuable time which he has thrown away in searching for errors in the works of others, which, devoted to his own, would have rendered it of more value to medical and statistical science.

